# Protective Effect of Celecoxib on Early Postoperative Cognitive Dysfunction in Geriatric Patients

**DOI:** 10.3389/fneur.2018.00633

**Published:** 2018-08-07

**Authors:** Yangzi Zhu, Rui Yao, Yan Li, Congyou Wu, Lei Heng, Meiyan Zhou, Li Yan, Yan Deng, Zhe Zhang, Lei Ping, Yuqing Wu, Shengtao Wang, Liwei Wang

**Affiliations:** ^1^Department of Anesthesiology, Xuzhou Central Hospital, Xuzhou, China; ^2^Jiangsu Province Key Laboratory of Anesthesiology, Xuzhou Medical University, Xuzhou, China; ^3^Department of Anesthesiology, Suzhou Xiangcheng People's Hospital, Suzhou, China; ^4^Department of Anesthesiology, Xuzhou Tumor Hospital, Xuzhou, China; ^5^Department of Neurology, Xuzhou Central Hospital, Xuzhou, China; ^6^Department of Pain, Shandong Provincial Hospital Affiliated to Shandong University, Jinan, China

**Keywords:** celecoxib, postoperative cognitive dysfunction, postoperative pain, geriatric patients, cyclooxygenase-2 inhibitor

## Abstract

**Objective:** Inflammation plays a key role in the etiology and pathology of postoperative cognitive dysfunction (POCD). Cyclooxygenase (COX)-2 inhibitor celecoxib is used for the treatment of acute pain due to its potent anti-inflammatory and analgesic effects. Herein, we evaluated the effects of celecoxib on POCD in geriatric patients.

**Methods:** A total of 178 geriatric patients undergoing total knee arthroplasty were randomly divided into two groups and treated with celecoxib (group C) or placebo (group P). The levels of perioperative plasma COX-2, IL-1β, IL-6, TNF-α, neuron-specific enolase, and S100β were detected in all patients. The pain intensity was measured by numerical rating scale (NRS). A battery of 9 neuropsychological tests was performed pre-operatively and 1 week, and 3 months postoperatively. Patients, whose postoperative performance declined by ≧1 standard deviation as compared to each preoperative test score on ≧2 tests, were classified as POCD.

**Results:** A significant decrease in POCD incidence was found in group C as compared to group P on postoperative day 7 (12.3% vs. 34.1%; *p* < 0.05). POCD incidence did not differ between the two groups at the 3-month follow-up (8.8 vs. 9.7%). NRS scores at days 3 and 4 post-surgery were significantly lower in group C (*p* < 0.05). Patients in group C showed lower level of plasma COX-2, IL-1β, IL-6, TNF-α, and S100β as compared to group P postoperatively (*p* < 0.05).

**Conclusion:** These results demonstrated that celecoxib can decrease early POCD incidence after total knee arthroplasty in geriatric patients, which might be mediated by suppressing inflammation and acute postoperative pain caused by surgical trauma.

Registration: Chinese Clinical Trial Register, ChiCTR-IOR-16008168.

## Introduction

Postoperative cognitive dysfunction (POCD) is a relatively common complication in surgical patients, and associated with prolonged hospitalization, risk of withdrawal from work, and dependency on social transfer payments (1, 2). Although the pathophysiology of POCD remains largely unknown, inflammation is reported as the main cause (3, 4). Peripheral surgical trauma has been shown to influence inflammatory processes in the brain (5–8). Animal studies indicated that pro-inflammatory cytokines play a pivotal role in mediating surgery-induced neuroinflammation, triggering the activation of neurogliocytes and the concurrent endogenous production of pro-inflammatory cytokines (6, 7). Increased expression of pro-inflammatory cytokines results in performance deficits of hippocampus-dependent cognitive memory (5). Cyclooxygenase (COX) enzymes are the rate-limiting enzymes of prostaglandin E2 (PGE2) synthesis in the modulation of physiological and pathological processes. The two types of COX: COX-1 and COX-2 participate in normal physiological activities and inflammation, respectively (9, 10). Elevated COX-2 levels were found in multiple neuroinflammation-related neurodegenerative diseases, such as multiple sclerosis, amyotrophic lateral sclerosis, and Alzheimer's disease (11).

Celecoxib is a highly selective COX-2 inhibitor that provides anti-inflammatory and analgesic effects by decreasing prostaglandin formation. It attenuates inflammation in both peripheral and central tissues with minimal adverse reactions. Given the anti-inflammatory and analgesic effects, celecoxib might potentially alleviate POCD. In animal experiments, COX-2 inhibitors were found to be effective in the treatment of surgery-mediated neuroinflammation and cognitive decline (12, 13). Celecoxib is the only COX-2 inhibitor approved by the US Food and Drug Administration (FDA) for the relief of the signs and symptoms of osteoarthritis and rheumatoid arthritis, for the management of acute pain, and for the treatment of primary dysmenorrhea. However, whether it has preventive effects against POCD in clinical practice remains unclear.

This study was conducted to investigate the effect of celecoxib in POCD incidence in geriatric patients undergoing total knee arthroplasty (TKA). The effect of celecoxib on pro-inflammatory cytokine expression and numerical rating scale (NRS) of pain were assessed.

## Materials and methods

### Study design and participants

A prospective, randomized, double-blind, parallel-arm placebo-controlled trial was registered with the Chinese Clinical Trial Register (ChiCTR-IOR-16008168). The study protocol was approved by the Ethics Committee of Xuzhou Central Hospital. Written informed consent was obtained from all patients. The inclusion criteria were patients aged 65–75 years who underwent elective TKA under general anesthesia. Patients who fulfilled any of the following criteria were excluded: Mini-Mental State Examination (MMSE) score <23; peptic ulcer disease; cardiac-cerebral vascular disease; chronic obstructive pulmonary disease; neurological or psychiatric disorders; allergic reactions to NSAIDs; drug and alcohol abuse; hepatic and/or kidney dysfunction; BMI > 35; inability to communicate. Psychiatric disorders were assessed through medical history and doctor-patient communication. Drug and alcohol abuse was assessed by self-report and inquiring about patients' family numbers. Height and weight of every patient were actually measured for the compute BMI. The included patients were randomized to receive capsules containing 200 mg celecoxib (Celebrex, Pfizer Pharmaceuticals LLC, Puerto Rico, USA) and placebo (filled with amylum) every 12 h for 7 days from the day before operation, respectively. In order to minimize the difference of placebo effect between two groups, placebo capsules were filled with fixed amount of amylum, which are difficult to be distinguished from celecoxib capsules. Patients, anesthetists, and investigators involved in this study were blinded to the group assignments.

### Anesthesia and operation protocol

All patients were intravenously administered scopolamine 0.3 mg (Shanghai Harvest Pharmaceutical Co. Ltd., Shanghai, China). Anesthesia was induced with 0.1 mg/kg midazolam (Nhwa Pharma. Corporation, Xuzhou, Jiangsu, China), 2 mg/kg propofol (Sichuan Guorui Pharmaceutical Co., Ltd, Leshan, Sichuan, China), 0.6 μg/kg sufentanil (Yichang Humanwell Pharmaceutical Co., Ltd, Yichang, Hubei, China), and 0.2 mg/kg cisatracurium (Jiangsu Hengrui Medicine Co. Ltd, Lianyugang, Jiangsu, China). Anesthesia was maintained with remifentanil (Yichang Humanwell) 0.1–0.2 mg/kg/min and propofol, with the propofol infusion rate adjusted to maintain target Bispectral Index Score at 40–60. The tidal volume was adjusted to 8 ml/kg with a ventilatory frequency of 8–12 beats/min to maintain an end-tidal CO_2_ pressure (PETCO_2_) level of 30–40 mmHg. Heart rate, blood pressure and peripheral oxygen saturation (SpO_2_) were recorded continuously. Dezocine and tropisetron were used for patient-controlled analgesia (PCA) within 48 h post-surgery. Patients' NRS scores were evaluated daily for 1 week.

### Evaluation of cognitive function

The cognitive function was determined by administering several neuropsychological tests, including nine subscales before surgery, and at 1 week and 3 months post-surgery. The neurocognitive tests measured memory, attention, concentration and psychomotor skills. The tests included: the Mental Control and Digit Span (forward and backward) subtests of the Wechsler Memory Scale, Visual Retention and Paired Associate Verbal Learning subtests of the Wechsler Memory Scale, Digit Symbol subtest of the Wechsler Adult Intelligence Scale-Revised, Halstead-Reitan Trail Making Test (Part A), and Grooved Pegboard Test (favored and unfavored hand) (14). The standard deviation (SD) for each test was computed from the preoperative scores. According to the definition proposed by Newman for postoperative cognitive deficits, a patient whose postoperative performance declined by ≥1 SD as compared to each preoperative test score on ≥2 tests was classified as POCD (15).

### Laboratory measurements

Blood samples were collected at the following time points: preoperative (T0) and 12 h (T1), 24 h (T2), and 48 h (T3) post-surgery. Plasma samples were centrifuged at 4,000 rpm for 10 min and stored at −80°C. The plasma levels of IL-1β, IL-6, TNF-α, COX-2, neuron-specific enolase (NSE), and S100β were quantified using commercial enzyme-linked immunosorbent assay (ELISA) kits (Nanjing Jiancheng Biological Project Company, Nanjing, China). Biomarker standards and samples were added to the wells of assay plates and incubated for 1 h at 37°C. The standard diluent was added to the blank wells. The horseradish peroxidase-conjugated antibody (0.1 mL) was added to each well and incubated for 40 min at 37°C. Subsequently, the plates were washed four times with phosphate-buffered saline, and chromogen solution (0.1 mL) was added to each well. The plates were gently mixed and incubated for 20 min at 37°C in the dark. Then, stop solution (0.05 mL) was added to each well and the optical density determined at 450 nm using a microplate reader. The plasma concentrations of inflammatory biomarkers were calculated based on the standard curves generated using recombinant human biomarkers.

### Statistical analysis

Statistical analysis was performed using the SPSS 19.0 (SPSS, Chicago, IL, USA). The normal distribution was evaluated by the Shapiro-Wilk test. All quantitative data were normally distributed and presented as the mean ± SD. The scores of neuropsychological tests between the two groups were compared by repeated measures ANOVA. The intervention state was considered as the inter-subject factor test, and time of evaluation as intra-subject factor. The comparisons of other quantitative data between the two groups were performed using the Student's *t-*test. Qualitative variables were compared using the Chi-square or Fisher's exact test.

## Results

### Patient characteristics

**Figure 1:**
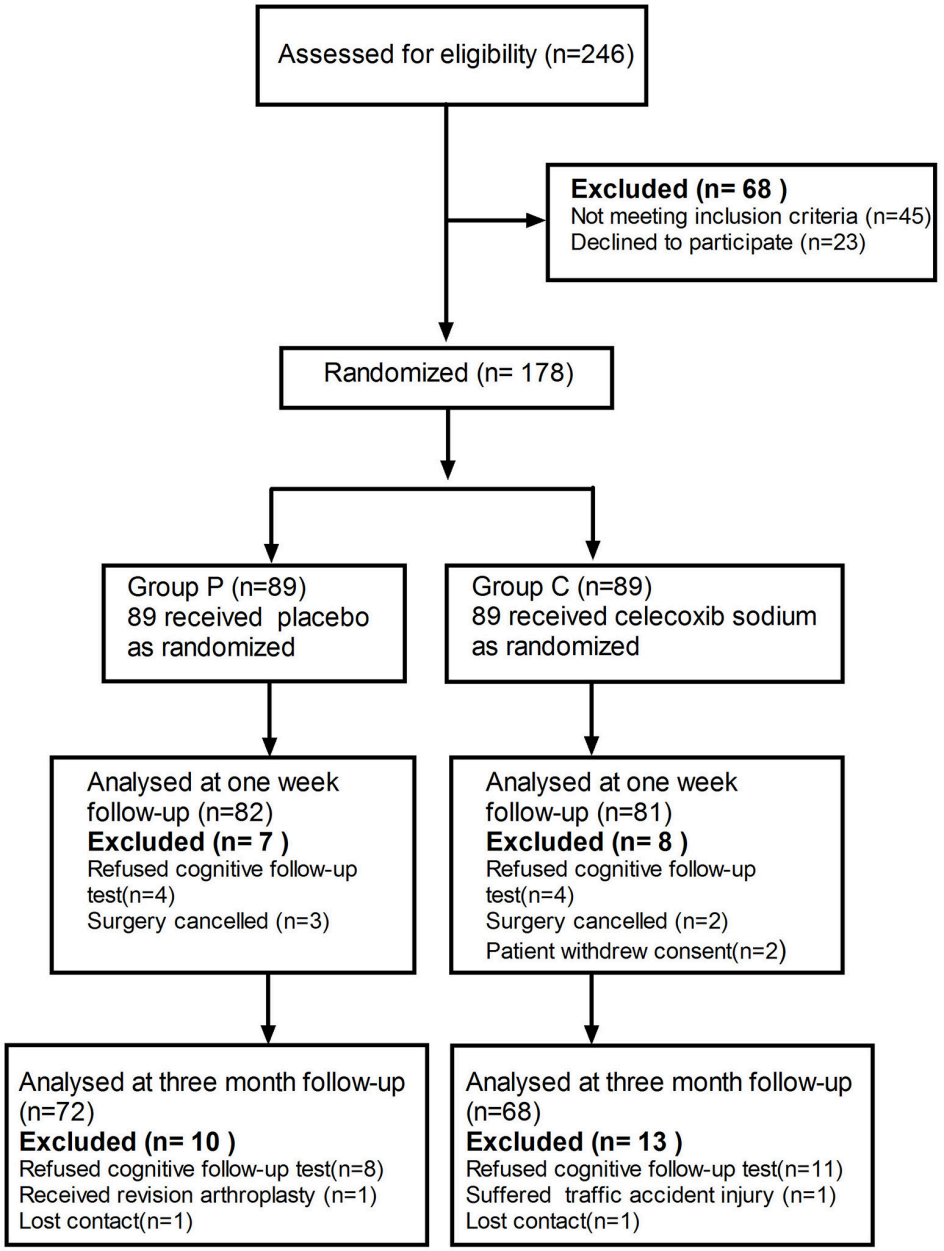
Schematic representation of enrollment of participants through each stage of our randomized trial.

A cohort of 178 patients, who completed the baseline assessment, were included in the trial. The exclusion criteria are listed in Figure 1. A total of 7 patients in the placebo group and 8 in group C (celecoxib treatment) were excluded before their 1-week cognitive follow-up appointment. Ten patients in the placebo group and 13 in group C were excluded before their 3-month cognitive follow-up appointment. No significant difference was observed in the basic demographic and clinical characteristics between the two groups (Table 1).

**Table 1:** Baseline characteristics of all participants.

**Admission characteristics**	**group P**	**group C**	***P*-value**
Age (years)	70.8 ± 5.2	71.4 ± 5.6	0.612
BMI (kg/m^2^)	27.3 ± 5.3	26.8 ± 4.9	0.551
Sex (M/F)	28/54	30/51	0.745
Education (year)	9.0 ± 3.2	9.2 ± 3.1	0.513
Hypertension	30 (37%)	28 (34.1%)	0.870
Diabetes mellitus	18 (22.2%)	20 (24.4%)	0.714
Smoker	13 (16%)	17 (21%)	0.425
MMSE scores	27.6 ± 2.7	28.1 ± 3.2	0.584
Length of surgery (min)	72 ± 11	69 ± 8	0.444
Estimated blood loss (ml)	257 ± 39	245 ± 41	0.412
Preoperative hospital stay (days)	2.6 ± 0.8	2.4 ± 0.6	0.625
Postoperative hospital stay (days)	7.9 ± 1.2	7.4 ±1.5	0.381
Postoperative nausea, vomiting	7 (8.6%)	9(11%)	0.610
Postoperative itchiness	5 (6.2%)	7 (8.5%)	0.565

*group C, celecoxib group; P, placebo group; BMI, body mass index; MMSE, Mini-Mental State Examination. Values are mean ± SD or number (percentages)*.

### Cognitive outcome

The mean and SD-values of the cognitive parameters in each group are shown in Table 2. No statistically significant time trend (intra-subject differences) was detected for any of the cognitive parameters. Furthermore, significant differences were observed between the two groups (inter-subject differences) in scores with respect to mental control, Digit symbol, and Pegboard favored hand. As compared to the celecoxib group, the placebo group showed a significantly decreasing trend (group time interaction) in most of the neuropsychological tests. 10/81 patients in the celecoxib group and 28/82 patients in the placebo group fulfilled the diagnostic criteria for POCD at 1-week follow-up (12.3 vs. 34.1%; *p* < 0.05). At the 3-month follow-up, no significant difference was detected in the POCD incidence between the two groups (8.8 vs. 9.7%; Table 3).

**Table 2:** Neuropsychological assessment scores at baseline, 7 days, and 90 days follow-up in patients.

**Variables**	**Group**	**Baseline**	**After operation 7th day**	**After operation 90th day**	***p*****-value**		
					**Time**	**Group**	**Time·Group**
Mental control	P	83.42 ± 11.9	70.1 ± 11.3	75.9 ± 13.8	0.383	0.023	0.034
	C	82.7 ± 13.2	78.5 ± 9.1	80.9 ± 12.1			
Visional rational	P	9.6 ± 3.1	8.4 ± 2.4	9.3 ± 2.8	0.496	0.163	0.042
	C	9.7 ± 3.8	9.5 ± 3.2	9.6 ± 3.1			
Paired associate verbal learning	P	18.0 ± 2.6	15.4 ± 3.0	16.9 ± 3.1	0.528	0.187	0.048
	C	17.8 ± 2.3	16.6 ± 2.8	17.2 ± 2.9			
Digit span forward	P	7.6 ± 1.6	7.1 ± 1.3	7.5 ± 1.0	0.623	0.309	0.413
	C	7.4 ± 1.4	7.2 ± 1.1	7.3 ± 1.5			
Digit span backward	P	4.3 ± 1.3	4.0 ± 1.7	4.2 ± 1.4	0.788	0.462	0.371
	C	4.4 ± 1.2	4.12 ± 1.5	4.3 ± 1.78			
Digit symbol	P	29.1 ± 7.3	16.9 ± 6.3	24.2 ± 10.4	0.259	0.031	0.040^*^
	C	28.6 ± 6.6	24.8 ± 8.4	27.1 ± 9.4			
Trails A	P	138.2 ± 41.3	117.8 ± 45.1	130.8 ± 35.9	0.374	0.259	0.116
	C	136.9 ± 37.6	126.2 ± 38.1	134.8 ± 38.4			
Pegboard favored hand	P	84.8 ± 10.3	69.4 ± 11.0	76.2 ± 8.9	0.176	0.039	0.043
	C	85.1 ± 9.4	83.2 ± 10.1	83.9 ± 10.3			
Pegboard unfavored hand	P	86.4 ± 11.9	81.2 ± 12.7	84.4 ± 13.4	0.417	0.742	0.303
	C	85.9 ± 12.1	83.6 ± 10.5	84.5 ± 11.8			

*group C, celecoxib group; P, placebo group; Data presented as mean ± SD, the P > 0.05, there were no statistical difference*.

**Table 3:** Comparison of occurrence of postoperative neuropsychological deficit.

**Number of deficits**	**After operation 7th day**		**After operation 90th day**	
	**Group P**	**Group C**	**Group P**	**Group C**
1	38	30	15	11
2	23	8	6	5
3	4	2	1	1
4	1	0	0	0
≥5	0	0	0	0
POCD patients (those with 2 or more deficits)	28 (34.1%)	10 (12.3%)^a^	7(9.7)%	6(8.8%)

a Comparison of the celecoxib group and placebo group, p < 0.001

### Postoperative pain and laboratory outcome

**Figure 2:**
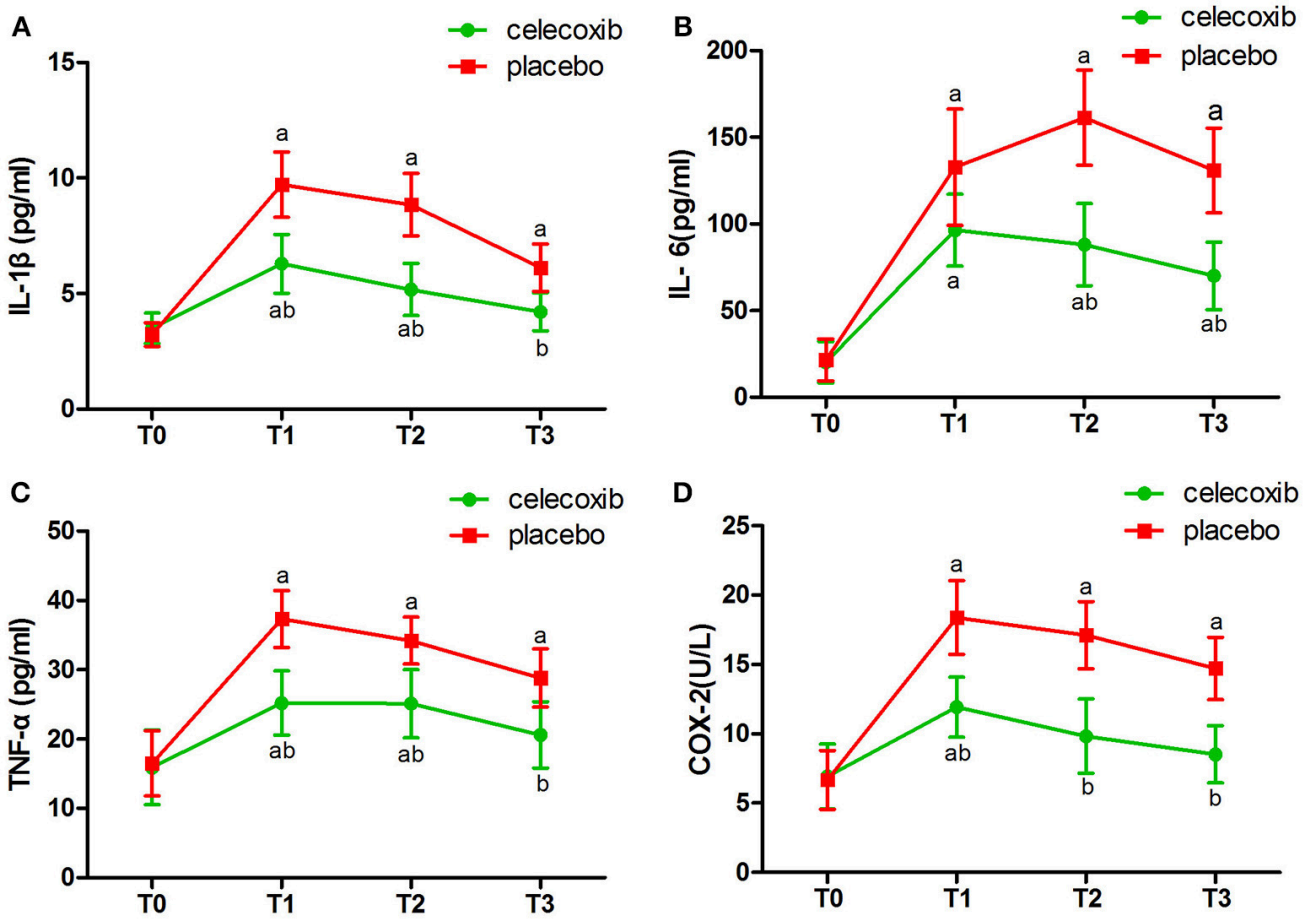
Plasma levels of IL-1β **(A)**, IL-6 **(B)**, TNF-α **(C)** and COX-2 **(D)** before and after surgery in celecoxib and placebo group. ^a^*P* < 0.05 vs. baseline (T0), ^b^*P* < 0.05 vs. placebo group.

**Figure 3:**
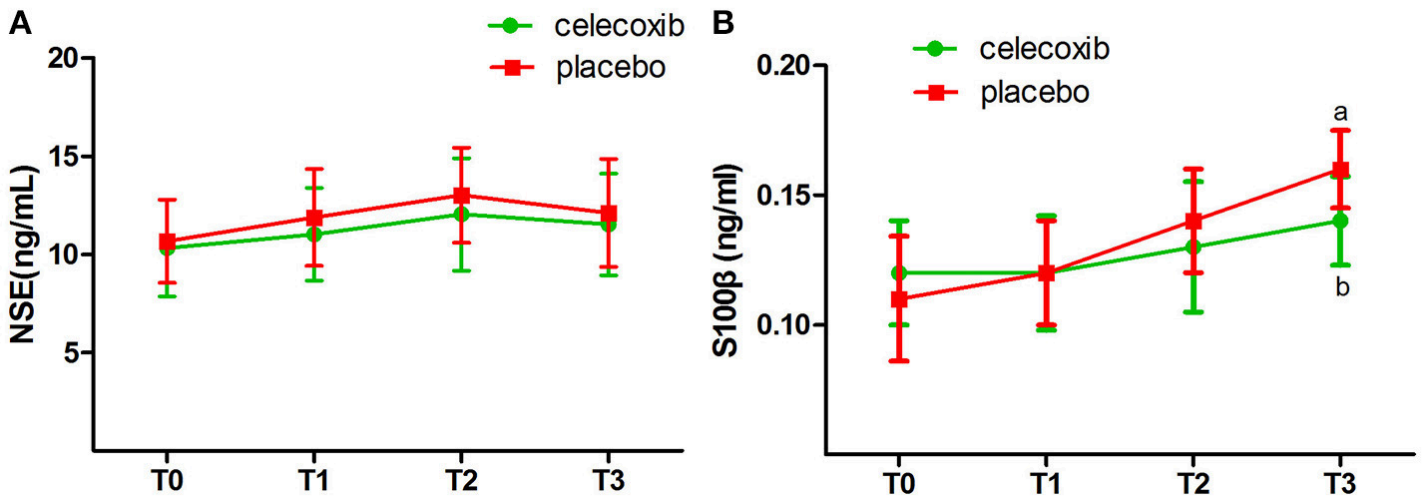
Plasma biomarkers of the neuronal damage before and after surgery in celecoxib and placebo group. NSE, neuron-specific enolase. **(A)** The levels of NSE in the plasma rise slightly after the operation, but there was no difference between two groups in NSE level. **(B)** The S100β levels in celecoxib group were lower than that in placebo group after the operation. NSE, neuron-specific enolase. ^a^*P* < 0.05 vs. baseline (T0), ^b^*P* < 0.05 vs. placebo group.

NRS scores in group C were significantly lower than in both groups at the day 3 and 4 postoperatively (Table 4). IL-1β ELISA showed that TKA increased the levels of IL-1β at day 2 postoperative in groups C and P; however, the levels in group C were markedly lower than that in group P at each time point post-surgery (*p* < 0.05, Figure 2A). The changes in the levels of IL-6, TNF-α and COX-2 in the two groups were similar to those of IL-1β (Figures 2B–D). The levels of NSE and S100β in the plasma rise slightly after the operation. The S100β levels in group C were lower than that in group P (Figures 3A, B).

**Table 4:** Numerical rating scale (NRS) pain scores.

**Postoperative days**	**Group P**	**Group C**	***P*-value**
1	1.8 ± 0.6	1.6 ± 0.7	0.483
2	1.7 ± 0.8	1.8 ± 0.8	0.562
3	3.5 ± 0.9	2.3 ± 0.7	0.021^a^
4	3.2 ± 0.8	2.2 ± 0.6	0.041^a^
5	2.3 ± 1.1	1.9 ± 0.9	0.181
6	1.6 ± 0.7	1.4 ± 0.8	0.425
7	1.2 ± 0.5	1.1 ± 0.6	0.617

*Data presented as mean ± SD*.

a *Signifcant between-group difference (group P vs. group C)*.

## Discussion

In the present study, we assessed the effect of celecoxib on postoperative cognitive dysfunction (POCD) in geriatric patients. The patients who received celecoxib had a significantly lower incidence of POCD as well as lower levels of plasma IL-1β, IL-6, TNF-α, COX-2, and S100β post-surgery as compared to the patients who received placebo. Moreover, celecoxib provided satisfactory analgesic efficacy after termination of patient-controlled analgesia (PCA). To the best of our knowledge, this is the first randomized clinical trial that investigated the protective effect of celecoxib on POCD and the underlying mechanisms.

Surgical injury activates the immune system resulting in peripheral inflammatory response. Pro-inflammatory cytokines, such as TNF-α, could readily penetrate the blood-brain-barrier (BBB) leading to neuroinflammation (16). S100β, a marker of increased BBB permeability (17), rise after the operation suggest BBB permeability is increased following TKA. But it is not known whether increased BBB permeability is due to structural or functional evolution in this study. More specific markers of the BBB vulnerability deserve further research. Increasing evidence demonstrated that neuroinflammation triggered by surgery plays a pivotal role in the development and progression of POCD (6, 7). High concentrations of pro-inflammatory cytokines disrupt neurological function and cause neuronal damage (18). Cyclooxygenase (COX) enzymes can induce cytokine production and enhance the permeability of BBB (19). Since celecoxib can easily span across the BBB (20), COX-2 in the central nervous system and other tissues will be inhibited by celecoxib. Animal studies showed that COX-2 inhibitors attenuated neuroinflammation and cognitive dysfunction caused by surgery. The current data also demonstrated that celecoxib effectively decreased levels of plasma COX-2 and pro-inflammatory cytokines after surgery.

Unlike any other COX-2 inhibitors, celecoxib can exert its anti-inflammatory effect through a COX-2-independent pathway. The infiltration of the inflammatory cells into the central nervous system and the expression of adhesion molecules, P-selectin, and intercellular adhesion molecule-1, were inhibited by celecoxib in COX-2-deficient mice (21). The wide spectrum of anti-inflammatory functions of celecoxib is beneficial in to mitigating the neuronal damage. We measured the levels of the plasma biomarkers pre- and postoperatively in all patients. NSE and S100β are closely related to neuronal damage and cognitive function. The present study showed that the plasma levels of NSE and S100β rise slightly after the operation, and a statistically significant difference was observed in the S100β levels between the patients who received celecoxib and those who received placebo at 48 h after operation. However, the reduction effect of celecoxib on NSE and S100β levels in the plasma is not remarkable, which might be attributed to the mild neuronal damage induced by arthroplasty. In addition, the plasma levels of NSE and S100β may be much lower than that in the cerebrospinal fluid. We did not withdraw cerebrospinal fluid in the current study.

Since pain results in neuroinflammation (22), postoperative pain might be critical for facilitating POCD. A recent study showed that satisfactory analgesia can decrease the incidence of POCD following total knee arthroplasty (TKA) in geriatric patients. (23) Animal studies demonstrated that postoperative pain might cause memory deficits (24), and such effects were reversed by a non-steroidal anti-inflammatory drug via its analgesic effects in animals (25). In the present study, celecoxib did not show an additional advantage of analgesia within 48 h post-surgery due to the efficiency of PCA. Yet PCA for 48 h is insufficient for TKA. A majority of the patients still needed pain relief on the third and fourth day after TKA. Thus, the NRS scores in group P were increased and the abirritation of celecoxib in group C became obvious when PCA was over. The postoperative pain decreased naturally due to wound healing 5 days post-surgery. The current data demonstrated that celecoxib could compensate for the gap in postoperative analgesia at the 3rd and 4th day post-operation. These findings indicated that the effective postoperative analgesia with celecoxib may reduce POCD incidence in geriatric patients.

Nevertheless, this study had several limitations. First, 21.3% loss of patients to 3-month follow-up may have resulted in statistical limitations in POCD incidence at the 3-month follow-up. Second, the population in this study is relatively narrow due to strict inclusion and exclusion criteria. Patients with psychiatric or neurological disorders, such as depression or insomnia, were excluded, to decrease the likelihood that the disease itself and drugs (i.e. benzodiazepine, antidepressants etc.) would interfere with evaluation of cognitive function (26, 27). However, depression and insomnia are not uncommon in geriatric patients with osteoarthritis of knee joint (28, 29). The evaluation of postoperative cognitive function and corresponding drug treatment should be further investigated in older adults with comorbid neuropsychiatric disorders and osteoarthritis. Third, inflammatory mediators in plasma directly reflect the systemic inflammation rather than neuroinflammation. Still, the plasma levels of S100β rise after the operation suggest at least partly BBB permeability is increased following TKA. Animal studies also indicated that peripheral inflammatory cytokines could penetrate the BBB to activate neurogliocytes. To some extent, the plasma levels of pro-inflammatory cytokines may reflect neuroinflammation. Although cerebrospinal fluid might be more suitable than plasma to evaluate neuroinflammation, it is restricted by ethical issues. Finally, compared to the cerebrospinal fluid, plasms might not be optimal for the detection of NSE and S100β. Thus, additional studies are needed to address these issues.

In conclusion, this study suggested that celecoxib alleviated POCD in geriatric patients. Anti-inflammatory and analgesic effects of celecoxib are critical for preventing POCD in geriatric patients. Future studies with large sample size are required to confirm the preventive effect of celecoxib on POCD.

## Author contributions

YZ, RY, and YL formulated the design of the studies, carried out the execution and analysis of the studies, and drafted the manuscript. CW, LH, MZ, LY, YD, ZZ, and LP were involved in data collection. YW, SW, and LW conceived the study, completed its design and coordination, and secured funding for the project.

### Conflict of interest statement

The authors declare that the research was conducted in the absence of any commercial or financial relationships that could be construed as a potential conflict of interest.
